# An update on the use of biologic therapies in the management of uveitis in Behçet’s disease: a comprehensive review

**DOI:** 10.1186/s13023-017-0681-6

**Published:** 2017-07-17

**Authors:** Thomas W. McNally, Erika M. Damato, Philip I. Murray, Alastair K. Denniston, Robert J. Barry

**Affiliations:** 1 University of Birmingham Medical School, University of Birmingham, Birmingham, UK; 2 Behcet’s Centre of Excellence, City Hospital, Sandwell & West Birmingham Hospitals NHS Trust, Birmingham, UK; 3 Birmingham & Midland Eye Centre, City Hospital, Sandwell & West Birmingham Hospitals NHS Trust, Birmingham, UK; 4 University of Birmingham Academic Unit of Ophthalmology, Birmingham & Midland Eye Centre, City Hospital, Sandwell & West Birmingham Hospitals NHS Trust, Birmingham, UK; 5 Centre for Translational Inflammation Research, School of Inflammation and Ageing, College of Medical and Dental Sciences, University of Birmingham, Queen Elizabeth Hospital, University Hospitals Birmingham NHS Foundation Trust, Birmingham, UK; 6 Department of Ophthalmology, Queen Elizabeth Hospital, University Hospitals Birmingham NHS Foundation Trust, Birmingham, UK

**Keywords:** Behçet’s disease, Biologic therapy, Health economics, Immunosuppression, Quality of life, Steroid-sparing agents, Uveitis

## Abstract

Behçet’s disease (BD) is a systemic vasculitis characterised by a relapsing remitting course, affecting multiple organ systems. In the eye, it is a cause of potentially blinding inflammation in the form of uveitis. Management of uveitis in BD often requires the use of systemic immunosuppression, in order to reduce disease activity and prevent accumulation of irreversible damage. Whilst corticosteroids remain the mainstay of treatment, long-term use is limited by the development of adrenocorticotrophic side effects. There has therefore been significant interest in the use of corticosteroid-sparing immunosuppressive agents, and more recently, biologic therapies. Recent publications have demonstrated biologic therapy to have beneficial effects both on overall disease control, and quality of life for patients with BD. Widespread use of such agents is however limited, partly by the lack of high quality research evidence, and partly by the prohibitive cost of biologic treatments. In this review, we discuss the most recent research investigating the use of biologic therapy in uveitis due to BD, with consideration of health economics and quality of life outcomes.

## Background

Behçet’s disease (BD) is a multi-system inflammatory disorder of unknown aetiology [1, 2]. Presentation is variable, depending on the organ system involved and the severity of the disease in each anatomical location [3]. BD is characterised by relapsing and remitting episodes of inflammation and may present with ocular manifestations, genital and oral apthae, gastrointestinal involvement, skin lesions, neurologic disease, arthropathy, and pulmonary, renal and vascular disease [4, 5]. No diagnostic test exists for BD, and accurate diagnosis is therefore dependent on identification of clinical features in accordance with internationally accepted diagnostic criteria [6]. The manifestations of the disease are largely attributable to widespread vasculitis [7].

BD occurs throughout all geographic locations, being most prevalent and often more severe in countries distributed along the ancient ‘Silk Route’ [8]. For example, in the UK BD is considered an orphan disease, with an estimated prevalence of 0.64 per 100,000, whilst prevalence increases to between 20 and 421 per 100,000 in Mediterranean and Chinese populations [9]. BD typically presents between 20 to 40 years of age; onset under the age of 25 years tends to be associated with an increased risk of ocular disease [7]. The condition is reported to be most aggressive in Far-Eastern young males [10–13]. Although BD has traditionally been considered sporadic there have been cases of familial clustering, suggesting a potential genetic predisposition to disease [14, 15]. Genetic anticipation, where the disease presents earlier in subsequent generations, is also evident [16].

Evidence suggests that the pathogenesis of BD involves an abnormal immune response following exposure to an exogenous agent, possibly infective, in patients who are genetically predisposed to the disease [17]. Several disease mechanisms have been proposed, with significant evidence supporting the involvement of human leukocyte antigen B51 (HLA-B51) [18–26]. More recently, there has been interest in the possibility of associations between BD and gut microbiota profiles. A particularly interesting association has been observed with reduced butyrate production, which is thought to be important in T-regulatory cell development [27].

Non-infective uveitis is rare in the general population, affecting 25–50 people per 100,000 in the UK. However, it is the most common ocular manifestation of BD and can involve the anterior, intermediate and posterior uveal tract or either in isolation, or in combination as panuveitis. Whilst there are no pathognomonic features, uveitis in BD typically presents with acute onset hypopyon and occlusive retinal vasculitis, with predominant inflammation of retinal veins rather than arterioles. Whilst uveitis due to BD is typically bilateral, flares of inflammation usually occur unilaterally and alternate between eyes [28]. Uveitis is associated with a worse visual outcome in males with BD [29, 30] and affects between 50% and 90% of BD sufferers depending on the geographic location of the population [31]. Up to 30% of patients with uveitis due to any cause experience significant visual impairment or legal blindness [32]. In addition to a reduced visual acuity patients can also suffer from decreased contrast sensitivity, increased light sensitivity, loss of depth and colour perception, floaters, glare and secondary glaucoma with loss of visual field. Uveitis, therefore, has a significant impact on quality of life [33].

In a cross-sectional study of 880 patients with BD, 30.9% and 24.2% of eyes in males and females respectively had a potential visual acuity of 0.1 LogMAR or less. It was predicted that the risk of loss of useful vision in 5 years was 21% in males and 10% in females, increasing to 30% and 17% respectively after 10 years [34].

Given the variability in presentation and severity, there are no widely-accepted ‘gold-standards’ in the treatment of BD. Furthermore, access to treatment varies by geographic location, often limiting the range of treatment options available. Disease management is therefore variable, with therapeutic options ranging from symptomatic relief through to systemic immunosuppression. Treatment is usually instigated and monitored by a multi-disciplinary team, requiring collaboration between dermatologists, ophthalmologists and rheumatologists, with input from cardiologists, genitourinary physicians and neurologists depending on presenting features.

In the following review, we consider the evidence available to guide management of uveitis in BD, with particular attention to biological agents. Until very recently, there has been a paucity of high quality evidence to support the use of biologic therapies in BD. However, outcomes of larger multicenter trials have recently been reported, providing an increasing quantity of convincing evidence for the benefit of biologic over traditional therapies. We wished to review existing and emerging literature in this field, and offer a clinical update in the biologics for the control of Behçet’s uveitis.

### Overview of current treatment strategies

Before considering recent advances in biologic therapy, it is necessary to review existing management strategies, to highlight both the strengths and weaknesses of current practice. Since BD is of unknown aetiology, treatment cannot be targeted at a specific causative agent. Thus, disease must be controlled via suppression of the immune response. Treatment of uveitis in BD typically relies on the use of corticosteroids for acute exacerbations, with other immunosuppressive agents introduced to achieve long-term control.

Initial treatment of uveitis in BD depends on both the severity and location of inflammation, highlighting the importance of precise diagnosis [33]. The aim of treatment is to control the inflammatory process in order to preserve sight. For anterior uveitis, topical corticosteroids combined with a cycloplegic agent are frequently used as a first line therapy. For posterior segment disease, or recalcitrant anterior uveitis, systemic therapy or local steroids are often required. Whilst systemic corticosteroids are commonly prescribed, long-term use is limited by the development of unwanted side effects and a resulting detrimental impact on quality of life. Potential side effects include weight gain, hypertension, osteoporosis, mood disturbance and glucose intolerance [33]. Despite these limitations, corticosteroid therapy remains the mainstay of acute management, owing to the rapid onset of immune suppression [35]. Unfortunately, disease activity often recurs on cessation of therapy; adjunctive immunosuppressive therapy is therefore used alongside corticosteroids to enable reduction of corticosteroid dosage, minimize adrenocorticotrophic side effects, and hopefully reduce relapse rates [36]. Such therapies are often referred to as “steroid sparing agents” (SSA).

Numerous SSA have been used to treat BD, each with varying cellular and biochemical targets. Whilst these agents are free of the aforementioned adrenocorticotrophic effects, they each have potentially serious side effects and require close monitoring [37, 38]. Table 1 summarises conventional systemic immunosuppressive agents used to treat non-infectious uveitis.

**Table 1:** A summary of conventional systemic immunosuppressive agents used to treat non-infectious uveitis

Medication	Mechanism	Typical dosage	Adverse effects	Price of typical one-year course of treatment for Behçet’s uveitis (£GBP)^a^
T-cell inhibitors				
Cyclosporine	T-cell function inhibitor	2.5–10 mg/kg/day PO in 2 divided doses	Infections, nephrotoxicity, hypertension, hirsutism and gingival hyperplasia	1111.43–4130.59
Tacrolimus	T-cell function inhibitor	0.15–0.30 mg/kg/day PO	Infections, nephrotoxicity, hypertension, diabetes mellitus and electrolyte imbalance	5468.43–7358.40
Anti-metabolites				
Methotrexate	Dihydrofolate reductase inhibitor	7.5–25 mg/week PO, SC, or IM	Infections, hepatotoxicity, oral ulcers, fatigue, alopecia, bone marrow suppression, pneumonitis, fetal loss and gastrointestinal disturbance	2271.36–4680.00
Azathioprine	Purine metabolism inhibitor	1–4 mg/kg/day PO	Infections, hepatotoxicity, fatigue, bone marrow suppression, hypersensitivity and gastrointestinal disturbance	120.34–174.98
Mycophenolate mofetil	Inosine monophosphate dehydrogenase inhibitor	500–1500 mg PO twice daily	Infections, bone marrow suppression, and gastrointestinal disturbance	620.50–1861.50
Leflunomide	Dihydroorotate dehydrogenase inhibitor	100 mg PO daily [×3 days], then 20 mg PO daily or every other day	Infections, bone marrow suppression, diarrhoea, hypertension and fetal loss	373.27–746.54
Alkylating agents				
Chlorambucil	Alkylates nucleic acid	0.1–0.2 mg/kg/day PO	Infections, bone marrow suppression, increased risk of malignancy, and sterility	2365.78–4731.57
Cyclophosphamide	Alkylates nucleic acid	1–3 mg/kg/day PO	Infections, bone marrow suppression, hemorrhagic cystitis, increased risk of malignancy, sterility, and alopecia	1014.70–2029.40
Other				
Colchicine	Inhibits neutrophil motility	0.5–2 mg/day PO	Infections, peripheral neuropathy, bone marrow suppression, sterility and alopecia	265.54–1062.15

^a^ Prices calculated according to recommended maintenance dose for one year of therapy for a 70 kg patient as prescribed and tested in up to date literature. Price as per BNF 70 [*Joint formulary committee. British National Formulary. BNF 70 ed. London: BMJ Group and Pharmaceutical Press; September 2015]*

### Biological therapies

Biological therapies, also known as biologic response modifiers, have many potential advantages for the treatment of uveitis in BD, particularly when traditional SSA therapy fails or there is intolerance to medication. Biologic agents are manufactured using recombinant DNA technology, designed with a detailed molecular understanding of the pathogenesis of the immune response. They enable more targeted immune modulation and therefore tend to have a more favorable side-effect profile, while offering a greater efficacy. Biologic therapy may take the form of monoclonal antibodies, cytokines, cytokine antagonists or soluble receptors [33].

Much of the research to date employing biologic agents for the treatment of uveitis has studied tumour necrosis factor alpha (TNF- α) inhibitors, which have been shown to significantly improve the outcome of uveitis in BD. Other biologic therapies are being developed to target different aspects of the disease pathogenesis including interleukin-1 (IL-1) and interleukin-6 (IL-6) [39–41]. In addition, the use of interferons - particularly interferon-α (IFN-α) - has shown promising outcomes in the management of uveitis in BD [42]. Current biologic therapies available for use in Behçet’s uveitis are summarized in Table 2, and the available evidence discussed throughout the following sections [43–48].

**Table 2:** A summary of selected biologics used to treat uveitis in Behçet’s Disease, their targets, doses, routes of administration and side effects

Agent	Target	Route of administration	Typical dosage	Adverse effects	Price of typical course of treatment for Behçet’s uveitis (£GBP)^a^
Tumour Necrosis Factor (TNF) inhibitors [41]					
Infliximab	TNF-α	Intravenous	Loading course of 3 × 3-5 mg/kg doses at 2-week intervals, followed by maintenance doses of 5-10 mg/kg at 4-week intervals [38]	Heart failure (congestive), infections, (particularly reactivation of tuberculosis), malignancy, thromboembolism, lupus-like disease, hypersensitivity reactions, neoplasia	80,776.85
Adalimumab	TNF-α	Subcutaneous	40 mg injection at 2-week intervals [38]		15,476.53
Etanercept	TNF-α, β	Subcutaneous	25 mg subcutaneously twice weekly [42]		9295.00
Golimumab	TNF-α	Subcutaneous	50 mg every month [38]		9115.64
Specific receptor antagonists					
Canakinumab	IL-1β	Intravenous or subcutaneous	150 mg at 4–8 week intervals [39]	Infections, nausea, abdominal discomfort	119,133.60
Tocilizumab	IL-6 receptor	Intravenous	4-12 mg/kg at 2–4 week intervals [38, 43–45]	Infections and hypersensitivity reactions	1638.40
Anakinra	IL-1 receptor	Subcutaneous	1 mg/kg/day [38]	Infections, injection-site reaction, headache, fever and gastrointestinal disturbance	4466.14
Gevokizumab	IL-1β	Intravenous or subcutaneous	0.3 mg/kg single infusion [38]	Infections and hypersensitivity reactions	n/a^b^
Lymphocyte Inhibitors					
Rituximab	B-cells via CD20	Intravenous	2 doses of 1 g 15 days apart [46]	Infections, muscular spasms, gastrointestinal discomfort, headaches and cardiovascular events	3492.60
Interferons					
Interferon α	Non-specific	Subcutaneous	6–9 MIU/day for 7 days, tapered down to 3 MIU 3 times a week and then discontinued [47]	Flu-like symptoms, bone marrow suppression, injection-site reaction	4132.20

^a^Prices calculated according to recommended standard treatment dose and duration of course for a 70 kg patient as prescribed and tested in up to date literature. Price as per BNF 70 [*Joint formulary committee. British National Formulary. BNF 70 ed. London: BMJ Group and Pharmaceutical Press; September 2015]*

^b^No pricing is available

### Search strategy

A systematic online literature search was performed using the PubMed database, Medline, EMBASE and the Cochrane Central Register of Controlled Trials (CENTRAL) for all studies published before December 2016 combining the terms “therapy OR therapeutic OR treatment”, “behçet*” (exploded), and all publication types relating to clinical trials as listed in the PubMed database. Abstracts were manually reviewed by two authors (RB and TM) and all papers reporting outcomes of biologic therapies were identified. To be considered for inclusion, all documented cases of BD must have been diagnosed according to the International Study Group (ISG) guidelines (1990) [7], or for those studies recruiting patients prior to the publication of these guidelines, diagnosis of BD must have been deemed concordant with ISG criteria both reviewing authors.

Publications were excluded from further review if the study did not report outcomes of biologic therapies, or did not specifically report outcomes for patients with BD. Duplicates, narrative reviews and editorials were excluded from further analysis. Due to the native language of the reviewers, we were unable to assess studies without an English language translation.

### Tumour necrosis factor – Alpha (TNF-α) inhibitors

#### Rationale for TNF blockade in Behçet’s disease

Inflammation in BD is considered to be mediated predominantly by T helper type 1 (Th1) lymphocytes, releasing cytokines such as Tumour Necrosis Factor (TNF) [49]. This is supported by the observation of increased numbers of monocytes and T lymphocytes expressing the gamma-delta receptor and increased levels of circulating TNF and soluble TNF receptors in the peripheral blood of patients with active disease [50–53]. Furthermore, high levels of TNF have been detected in the aqueous humor of patients with Behçet’s uveitis [54, 55].

There has therefore been significant interest in TNF blockade, with several agents developed to inhibit TNF signaling. Numerous targets have been identified in the signaling pathway for potential therapeutic modulation. These agents are discussed in further detail below.

#### Infliximab

There is considerable evidence accumulating to support the efficacy of infliximab (Remicade; Janssen Biotech, Inc., Horsham, PA, USA) for the treatment of BD. Infliximab is a chimeric monoclonal antibody directed against TNF and has been shown to be effective and fast acting in the treatment of Behçet-associated panuveitis [56–62].

Suhler et al. demonstrated the efficacy of infliximab in a non-comparative case series of 23 patients with refractory uveitis; four patients had BD, all of which had a diagnosis of BD-related panuveitis [58]. Patients received 3 infliximab infusions at 0, 2 and 6 weeks at a dose of 3 mg/kg if given alongside other immunosuppressive medications (*n* = 20), or at 5 mg/kg if infliximab was given as monotherapy (*n* = 3). Patients who had responded to treatment at week 10 were given a further infusion at week 14 (8 weeks after the loading schedule) and then every 8 weeks up to completion of the study at 50 weeks. Treatment success was assessed by four outcome measures, comprising end point visual acuity, control of intraocular inflammation, improvement in inflammatory signs on fluorescein angiography or optical coherence tomography, and ability to reduce other anti-inflammatory medications. Treatment was deemed successful if there was improvement in any one of these four subcomponents, in the absence of deterioration in any variable. According to these criteria, success was reported in 18 out of 23 patients at the 10-week follow up point.

All four patients with BD-related panuveitis showed improvement in at least two of the reported outcome measures, with two patients showing an improvement in three outcomes. However, only one patient with BD-related panuveitis demonstrated an improvement in visual acuity. This patient exhibited an improvement from 20/50 at week 0 to 20/30 at week 10 in both eyes. In addition, two patients with BD experienced significant adverse effects, although these were not severe enough to warrant discontinuation of treatment: one patient suffered recurrent vitreous haemorrhage that resolved on observation, another had one episode of nephrolithiasis that was treated in the emergency department and did not require admission. All patients with BD completed the study. In contrast, five patients with uveitis due to other causes were unable to complete the course of therapy due to adverse side effects, including recurrent infections, hypersensitivity reactions and heart failure.

Markomichelakis et al. reported the outcome of a comparative study assessing the efficacy of a single intravenous infusion of infliximab versus intravitreal triamcinolone, demonstrating that infliximab was not only better at reducing total ocular and fundus inflammation, but was also faster acting than corticosteroid therapy [60]. The prevalence of retinal vasculitis had reduced from 79% at baseline to 15% by 14 days follow-up in the infliximab group, compared to 100% and 87.5% respectively in the intravitreal triamcinolone acetonide group. Hamza et al. further demonstrated the safety and efficacy of a single injection of 1 mg/0.05 ml intravitreal infliximab in a series of 20 patients with refractory uveitis due to BD. By 18 weeks follow-up, they reported a statistically significant improvement in mean visual acuity, reduction in mean central macular thickness, and reduction in mean vitreous haze scores [62].

In 2008 the European League Against Rheumatism (EULAR) Committee published recommendations for the management of BD, in which they advocate the routine use of infliximab for patients with severe eye disease. Specifically, these recommendations state that any patient with BD-associated eye disease should initially be managed on a treatment regime that includes both azathioprine and systemic steroids, with the addition of either infliximab or cyclosporine A for patients with severe eye disease. Alternatively, interferon-α therapy can be used with or without corticosteroids [63].

Caution must however be exercised when using infliximab therapy due to potentially severe adverse side-effects (Table 2). A 2016 study found 28% of patients experienced side effects with 13% deemed ‘serious’, such as hypersensitivity reactions (*n* = 10), autoimmune disease (*n* = 6) and neoplasia (*n* = 4) [64]. These adverse effects are most likely due to the murine origin of the variable region of the molecule, and can be attenuated by concomitant anti-histamine and pain-relief medication. More significantly, there is an increased risk of developing disseminated TB, and there have also been reported cases of demyelinating disease in patients using TNF inhibitors; these risks are common to all anti-TNF agents [65]. Patients should therefore be screened for undiagnosed TB prior to commencing biologic therapy, and these agents should be used with caution in those at increased risk of demyelinating disease. Patients prone to recurrent opportunistic infections should be monitored closely and those with active infections should avoid therapy with this agent [39].

#### Adalimumab

Adalimumab (Humira; AbbVie, Inc., North Chicago, IL, USA) is a human-derived monoclonal antibody directed against TNF-α. It has predominantly been used when infliximab has proven unsuccessful, or when patients opt for subcutaneous infusions rather than intravenous injections; in both cases it has been demonstrated to be highly effective [66, 67]. A 40 mg injection once every two weeks has been shown to be well tolerated, however potential side effects including hypersensitivity reactions, infections or heart failure have been reported [39], in addition to the risks of TB and demyelination as discussed above. It has also been demonstrated as a successful first line treatment:

A 2010 study by Bawazeer et al. reported the outcome of 21 eyes of 11 male patients with uveitis due to BD, treated with adalimumab therapy [68]. Within four weeks of commencing therapy 10 of the 11 patients exhibited complete resolution of inflammation. Adalimumab was well tolerated in this series, with no patients experiencing any serious adalimumab-related side effects. This is most likely due to adalimumab being a human-derived preparation. In addition, adalimumab enabled dosages of concurrent immunosuppressive agents and corticosteroids to be reduced in many patients, and stopped completely in six and three patients respectively. Despite these promising results, it must be acknowledged that the study size was small and larger randomised controlled trials should be undertaken.

#### Etanercept

Etanercept (Enbrel; Immunex Corporation, Thousand Oaks, CA, USA) is a fusion protein of two p75 TNF receptors and an Fc molecule that blocks the action of TNF- α. Etanercept has primarily been investigated in the management of mucocutaneous and articular manifestations in patients with BD [43]. A twice-weekly 20 mg subcutaneous injection has previously been shown to be effective in the management of uveitis, as well as for mucocutaneous and gastrointestinal disease manifestations [44].

A number of case studies using etanercept in BD-associated uveitis have been reported [69–71]; the largest of these reported outcomes for 10 patients with severe uveitis in whom combination therapy with corticosteroid, azathioprine and cyclosporine-A had been ineffective [70]. Adding etanercept to the treatment regimen led to a reduction in ocular inflammation, improving visual acuity and allowing the corticosteroid dose to be reduced. However, after etanercept was stopped, uveitis returned in all patients within 6 months. Patients also suffered similar side effects to those experienced with other anti-TNF-α agents, which are summarized in Table 1. Paradoxically, etanercept-induced ocular inflammation has also been reported in non-BD cohorts. The underlying mechanism for this pro-inflammatory effect is not fully understood [72].

As a result of such observations, etanercept is not routinely used as a first-line agent in the management of BD-related uveitis: In a 2014 systematic review, Levy-Clarke et al. made recommendations for the use of anti-TNF biologic agents in patients with ocular inflammatory conditions. Infliximab and adalimumab were suggested as first line for patients with refractory BD-associated uveitis, and etanercept as second line owing to its lower success rates [73].

#### Golimumab

Golimumab (Simponi; Janssen Biotech, Inc.) is a monoclonal antibody to TNF-α that is administered subcutaneously once-monthly at a dose of 50 mg. Mesquida et al. reported a single case of Behçet’s associated uveitis successfully treated with golimumab injections [74]. In this case uveitis was refractory to other TNF-α inhibitors, but inflammation resolved after golimumab injections were commenced. In addition, the dosage of adjunctive cyclosporine-A was reduced to 150 mg/day, and Prednisone to 5 mg/day. After six months treatment the uveitis remained quiescent and the patient remained asymptomatic with 6/6 visual acuity. The side-effect profile of golimumab is similar to other anti-TNF- α therapy (Table 2).

In a more recent study, Santos-Gómez et al. demonstrated the efficacy of golimumab in four patients with BD-associated uveitis. This study reported outcomes of seven patients with refractory BD-associated uveitis in whom adalimumab and/or infliximab had been ineffective or poorly tolerated. Seven of 124 patients were treated with alternative biologic agents, of which four received golimumab, two received tocilizumab and one received rituximab. All seven cases achieved complete remission of uveitis at one year of follow-up. Furthermore, mean best-corrected visual acuity improved from 0.71 ± 0.24 LogMAR at baseline to 0.92 ± 0.13 LogMAR at three months follow-up (*p* = 0.03). Therapy was well tolerated with no serious side effects reported. The authors suggest that golimumab may therefore be effective in managing BD-associated uveitis that is refractory to standard therapies and other biologic agents [75].

### Specific receptor antagonists

#### Rituximab

Rituximab (Rituxan; Genentech, Inc., South San Francisco, CA, USA) is a monoclonal antibody to CD20, which acts through depletion of B-cells [76, 77]. There is limited published evidence to support its use for uveitis in BD.

Sadreddini et al. reported outcomes in a single patient with loss of vision due to retinal vasculitis resistant to prednisolone and azathioprine, who was treated successfully with rituximab, achieving 24 months of disease remission [78]. Davatchi et al. later performed a randomized, single blind pilot study involving 20 patients with retinal vasculitis resistant to cytotoxic drugs [79]. Patients were randomized to receive either two courses of rituximab at a dose of 1000 mg at 15-day intervals in combination with oral prednisolone (0.5 mg/kg/day) and methotrexate (15 mg/week), or combination therapy comprising cyclophosphamide (1000 mg/month), azathioprine (2-3 mg/kg/day) and prednisolone (0.5 mg/kg/day). The primary outcome was measured using the Total Adjusted Daily Activity Index (TADAI), showing a statistically significant improvement in TADAI score for patients receiving rituximab but not those on traditional combination therapy. No statistically significant difference was reported in improvement of retinal vasculitis between treatment groups, and both groups demonstrated a similar statistically significant improvement in macular oedema. Whilst this study suggests that rituximab may be superior to combination therapy in controlling overall disease activity, there is insufficient evidence to suggest that it is superior to combination therapy for control of intra-ocular inflammation.

In this series, two patients experienced conjunctivitis in the first week following rituximab infusion, one developed pneumonia and one developed herpes zoster, both four months following treatment. Mild infusion-related reactions were observed in two patients [79].

#### Tocilizumab

Evidence for the use of Tocilizumab (Actemr; Genentech, Inc.), a monoclonal antibody against the IL-6 receptor, is limited but encouraging [45–47, 80, 81]. In 2014 a study reported 3 women with Behçet’s uveitis who were resistant to immunosuppressive therapy and one anti-TNF biologic. Following treatment with intravenous tocilzumab, a reduction in ocular inflammation was observed in all patients, being sustained for a mean period of 7.3 months [45]. Other case reports also offer support for the use of Tocilizumab for recurrent or resistant BD at a dose of 4-12 mg/kg every 2–4 weeks [40, 45, 46, 80]. Reported side effects are relatively minor and include infections and hypersensitivity reactions.

#### Anakinra

Anakinra (Kineret; Swedish Orphan Biovitrum AB [publ], Stockholm, Sweden) is an IL-1 receptor antagonist and its use has only recently been reported in BD [82]. The drug prevents IL-1-mediated activation of the immune response. In a study by Cantarini et al. 9 patients with BD refractory to TNF inhibitors were treated with a 1 mg/kg daily subcutaneous injection of anakinra. Eight of nine patients displayed resolution of disease activity within 4 weeks of injection, and no adverse events were reported throughout the follow-up period. These results are especially promising since all 5 patients who began anakinra therapy specifically for management of BD-related uveitis demonstrated complete resolution of ocular inflammation. Further studies are required in this area.

#### Daclizumab

Daclizumab (Zenapax; Hoffman-La Roche Ltd., Basel, Switzerland) is a humanized monoclonal antibody to the alpha subunit of the IL-2 receptor on the surface of T-cells, administered intravenously, at a starting dose of 1 mg/kg once every 2 weeks, with dose and frequency titrated to response and side effects to a maximum of 200 mg [83]. It has been shown to be well tolerated by patients in the management of uveitis, with side effects including lymphadenopathy, psoriaform rashes, mild peripheral oedema and infections [84]. The data for efficacy of daclizumab in management of uveitis due to BD has been equivocal [85–88]; Buggage et al. completed a double-masked, randomized controlled trial, concluding that daclizumab was less effective than placebo in the management of ocular complications of BD [87]. Despite showing promise in the treatment of non-Behçet’s uveitis, daclizumab was discontinued by the manufacturer in 2009 due to decreasing market demand.

### Cytokine inhibitors

#### Canakinumab

Canakinumab (Ilaris; Novartis International AG) is a human monoclonal antibody against IL-1β. Canakinumab neutralizes IL-1β by competitively binding to the IL-1 receptor and consequently blocking the signaling by the antigen:antibody complex [89]. In a recent case report canakinumab was demonstrated to be effective in treating BD-associated panuveitis [90]. The patient was a 16-year-old female with severe bilateral panuveitis, with hypopyon and retinal vasculitis. The patient had been treated with other agents without success, including IFN-α, conventional corticosteroid therapy combined with immunosuppressants, infliximab, adalimumab and anakinra. However, a single subcutaneous injection of 150 mg canakinumab resulted in complete resolution of inflammation lasting 8 weeks with an associated improvement in visual acuity.

A more recent study by Fabiani et al. (2017) investigated the efficacy of both canakinumab and anakinra in treating BD-related uveitis [91]. A total of 31 affected eyes from 19 patients were treated with canakinumab, anakinra or both. For seven patients, IL-1 inhibitor therapy was their first exposure to biologic therapy. The remaining 12 patients had previously received other biologic agents. After 12 months of IL-1 inhibitor therapy the number of ocular inflammatory flares had reduced from 200 episodes/100 patients/year to 48.7 episodes/100 patients/year (*p* < 0.0001). The authors concluded that IL-1 inhibitor therapy is effective for managing refractory BD-related uveitis, providing long-term control of ocular inflammation.

#### Gevokizumab

Gevokizumab (XOMA 052; XOMA Corporation, Berkeley, CA, USA) is a monoclonal antibody against IL-1β. Gevokizumab reduces the binding affinity of IL-1β to its receptor by occupying an allosteric site on the IL-1β molecule, the resulting complex has reduced affinity for the IL-1 receptor [89]. A 98-day pilot study followed 7 patients with Behçet’s uveitis resistant to cyclosporine-A and azathioprine. A single 0.3 mg/kg infusion of gevokizumab resulted in complete resolution of intraocular inflammation within a median duration of 14 days (range 4–21 days), with a median duration of response of 49 days, with one patient remaining disease free for the full 97 days of follow-up [92].

In 2015 the results of a phase III, double-masked, placebo controlled trial studying the use of gevokizumab in Behçet’s uveitis were reported online [93]. This demonstrated a failure to achieve the primary outcome of increasing time to first exacerbation of ocular inflammation. Whilst the authors described promising secondary outcomes of an improvement in visual acuity and reduction in the overall number of uveitis exacerbations, there is currently insufficient evidence to support widespread use of gevokizumab in uveitis due to BD. To date, the results of this trial of not been published in a peer-reviewed format.

Few adverse reactions have been reported for gevokizumab, with infections and hypersensitivity reactions being the most common [94].

#### Secukinumab

Secukinumab (AIN457; Novartis International AG) is a human, monoclonal antibody against IL-17A [95, 96]. The SHEILD study was a randomised, placebo-controlled, multicentre phase III trial involving 118 patients with uveitis due to BD, in which secukinumab was administered subcutaneously at a dose of 300 mg 2- or 4- weekly [97]. The primary outcome was defined as reduction in uveitis recurrence or vitreous haze score on concomitant withdrawal of immunosuppressive therapy; unfortunately this was not achieved and as a result, secukinumab is not currently employed in the management of uveitis in BD.

### Interferons

#### Interferon-α

Evidence for the use of interferon (IFN) α-2a, a cytokine modulating the immune response, is promising. A treatment regimen of 6–9 MIU/day for 7 days, tapered down to 3 MIU 3 times a week and then discontinued according to treatment response has proven effective [48]. A systematic review of 32 original-reports and 3 selected abstracts between 1986 and 2002 has previously been published [98]; in this review, 182 patients with Behçet’s uveitis receiving IFN-α were identified, of whom 94% exhibited partial or complete remission of their intra-ocular inflammation. The review also demonstrated that higher doses of IFN- α (30.3 ± 31.7 × 106 IU (median, 24 × 10^6^; range 6–12 × 10^6^ IU) per week)) were associated with long-term remission of up to 56 months once treatment was discontinued, compared to lower doses (16.2 ± 28.8 × 10^6^ IU per week (median, 3 × 10^6^; range, 2.8–64 × 10^6^ IU)). Meta-analysis was limited due to variation in study design, however the authors concluded that there was significant support for the use of IFN-α treatment of uveitis associated with BD [99]. Other studies published since this review have also strongly supported the use of IFN- α in Behçet’s uveitis [48, 99–115].

Kotter et al. demonstrated the benefits of IFN-α in the management of both ocular and extra-ocular manifestations of BD [99]; this study followed 50 patients who were treated with IFN-α-2a. In affected eyes (*n* = 79) the mean visual acuity rose significantly from 0.56 at week 0 to 84.0 at week 24 (*P* < 0.0001). Of these 79 eyes, 37 remained stable after 108 weeks. 46 of the participants with ocular manifestations demonstrated a response to treatment, demonstrating a 92% success rate. This study also reported improvement in control of extra-ocular disease manifestations with IFN-α-2a therapy, and enabled concurrent corticosteroid dose to be reduced.

Two studies conducted by Deuter et al. have demonstrated an ability to achieve long-term remission of Behçet’s uveitis with IFN- 2α therapy [106, 107]: In their 2010 study of 53 patients (96 eyes) with Behcet’s uveitis, IFN-2α was initially administered at a dose of 6 million IU per day, being tapered to a maintenance dose of 3 million IU twice per week, and then discontinued according to treatment response. During a median follow up period of 6.0 years (range 2.0 to 12.6 years) visual acuity improved or remained stable in 91 of 96 eyes. Complete remission of ocular inflammation was demonstrated in 50% of patients 46 months after cessation of the first IFN-2α course. It was concluded that IFN-2α therapy is able to induce long-lasting remission of ocular-BD while also significantly improving visual prognosis [107].

Further evidence of long-term efficacy was demonstrated in a 2016 study by Kavandi et al. The authors reported on 8 patients whose visual acuity had improved or stabilised as a result of IFN-α-2a therapy, demonstrating that disease remained in remission with no adverse effects of therapy 2 years after IFN-α-2a discontinuation [114].

Interferon therapy has also been shown to enable reduction of concurrent corticosteroid dose. In a multicentre study by Lightman et al. outcomes of 72 patients were reported, demonstrating that the corticosteroid dose in patients receiving interferon therapy could be reduced to 6.5 mg/day compared to 10 mg/day in those receiving non-interferon therapy [115].

Support for the use of IFN-α to treat uveitis in BD is therefore increasing; current data reveals response rates of between 80% and 90% with a low relapse rate on cessation of treatment. In addition, the use of IFN-α allows oral steroid doses to be reduced, thus improving quality of life for the patient. Furthermore, IFN-α has also been shown to simultaneously improve other systemic manifestations of BD. However, potentially severe side effects such as flu-like symptoms, bone marrow suppression and injection-site reactions have been reported. Rarely, severe depression and suicidal ideation have also been reported [116]. Therefore, more clinical trials – ideally randomised, placebo controlled trials – must be carried out before an informed decision can be made about the routine use of IFN-α in uveitis due to BD.

### Effect of biologic therapies on quality of life in Behçet’s uveitis

Uveitis affects between 50% and 90% of BD sufferers depending on the geographic location of the population [41]. Up to 30% of patients with uveitis experience significant visual impairment or legal blindness [115]. In addition to a reduced visual acuity, patients may also suffer from decreased contrast sensitivity, increased light sensitivity, loss of depth and colour perception, floaters, glare and loss of visual field. Uveitis therefore has a significant impact on vision-related quality of life [36].

Biologic therapies have been shown to have a significantly positive impact on quality of life in patients suffering from uveitis due to BD. A study by Sakai et al. reported a positive impact on health-related quality of life (HR-QOF) and vision-related quality of life (VR-QOF) in patients with Behçet’s uveitis treated with infliximab [117]; twenty patients suffering from frequent uveitis attacks due to BD were asked to complete the EuroQol-5D questionnaire (EQ-5D) and the 25-item National Eye Institute Visual Function Questionnaire (NEI VFQ-25), before treatment and at 6 months and 12 months after treatment. The EQ-5D score improved from 0.66 ± 0.17 during the 6-month period prior to treatment to 0.97 ± 0.08 and 0.96 ± 0.07 at 6 and 12 months following treatment demonstrating a significant improvement in quality of life (*P* ≤ 0.0001). The authors also demonstrated improvements in general and mental health.

Lightman et al. also demonstrated an improvement in quality of life for patients on interferon therapy compared to standard therapy after 36 months follow-up [115]. The study found that interferon therapy allowed the tapering down of corticosteroid and immunosuppressive doses without an increase in relapse rate and alongside significant improvement in BD-related quality of life scores (*p* = 0.008).

Since there is no known cure for BD at present, the aim of the treating clinician should be to maximise function and maintain or improve quality of life for all patients. There is a growing body of evidence to suggest that biologic therapies may be an effective method of achieving both aims.

## Conclusion

The treatment of BD-associated uveitis, and the outlook for patients, has markedly improved over recent decades. With the advent of new technologies, biologic medications offer an exciting and effective therapy. Until recently, biologic therapies have been used mainly as an alternative treatment after immunosuppressive and corticosteroid therapies have failed, however accumulating evidence supports their use as first line agents. There are numerous benefits to the use of biologics, particularly with regard to quality of life and duration of treatment effect.

Whilst the benefits of biologic therapies compared to conventional immunosuppressive treatment are evident in terms of patient outcomes, their high cost may prove to be a limiting factor in their widespread adoption, with annual costs of biologic therapies often exceeding £100,000 (Table 2). Clinicians are increasingly having to make difficult decisions about whether to offer new and expensive biologic therapies, or to continue with more established agents that are cheaper due to the financial restrictions enforced by healthcare authorities [118].

Undoubtedly, large multicentre and well-designed studies are needed to develop further our understanding of both Behçet’s uveitis and biologic therapies, response rates and their long-term outcomes [1, 40]. It is hoped that further research will develop a biologic therapy that is universally effective, rapidly acting, has few side effects and is affordable, ultimately improving both clinical and quality of life outcomes for patients.

## Abbreviations

BD: Behçet’s disease
IFN: Interferon
IL: Interleukin
QoL: Quality of life
SSA: Steroid sparing agent
TNF: Tumour necrosis factor
